# Abuse, self-harm and suicidal ideation in the UK during the COVID-19 pandemic

**DOI:** 10.1192/bjp.2020.130

**Published:** 2020-07-13

**Authors:** Eleonora Iob, Andrew Steptoe, Daisy Fancourt

**Affiliations:** Department of Behavioural Science and Health, University College London, UK

**Keywords:** COVID-19, mental health, abuse, self-harm, thoughts of suicide or self-harm

## Abstract

This study explored patterns of abuse, self-harm and thoughts of suicide/self-harm in the UK during the first month of the COVID-19 pandemic using data from the COVID-19 Social Study (*n*=44 775), a non-probability sample weighted to population proportions. The reported frequency of abuse, self-harm and thoughts of suicide/self-harm was higher among women, Black, Asian and minority ethnic (BAME) groups and people experiencing socioeconomic disadvantage, unemployment, disability, chronic physical illnesses, mental disorders and COVID-19 diagnosis. Psychiatric medications were the most common type of support being used, but fewer than half of those affected were accessing formal or informal support.

The COVID-19 pandemic is presenting an unprecedented global challenge not just for infectious disease medicine but also for mental healthcare. Concerns about the psychological, social and neurological impact of the disease have been reported,^1^ along with preliminary data suggesting adverse effects on a range of aspects of mental health, including anxiety and depression.^2^

Self-harm, suicidal thoughts and abuse are already substantial problems in the UK. The prevalence of life-time experience of self-harming was reported to be 6.4% in 2014;^3^ prevalence of life-time thoughts of suicide was around 20.6%, with 5.4% reporting such thoughts in the previous year;^4^ and 7.5% of women and 3.8% of men reported experiencing abuse from a partner or family member in the year ending March 2019.^5^ But there have been rising concerns about people experiencing higher levels of psychological or physical abuse while social distancing at home during the COVID-19 pandemic, alongside increases in self-harming or suicidal ideation. Staying at home because of social distancing measures could increase the exposure of individuals to violence and abuse.^6^ Economic adversity and unemployment could lead to additional stress for individuals, especially disadvantaged and vulnerable groups.^7,8^ Individuals may be unable to access usual social networks that could provide support and may experience increased loneliness and isolation. Additionally, pressures on health services have led to disruptions to existing mental health services and reductions in use by individuals.^7^ All of these factors are linked with higher rates of self-harm, suicide and poor mental health outcomes.^9,10^

Already, some helplines have reported increases in the volume of calls since lockdown began and other data also suggest an increase in abuse-related incidents.^7^ However, data about the characteristics of people reporting such experiences in the UK during the COVID-19 pandemic are still scarce. Further, it is unclear whether people experiencing abuse and self-harming are managing to access any support. This is vital to understand in order to identify what further support is needed. Therefore, this study sought to address these evidence gaps by exploring patterns of abuse, self-harm and thoughts of suicide or self-harm in the UK in the first month of lockdown due the COVID-19 pandemic and exploring whether those having such experiences were accessing formal or informal mental health support.

## Method

We used data from University College London's (UCL's) COVID-19 Social Study, a large longitudinal study on the psychological and social experiences of over 50 000 adults in the UK during the pandemic. The study began on 21 March 2020, involving online questionnaires completed by participants on a weekly basis. For this analysis, we focused on participants recruited between 21 March and 20 April 2020. From a total sample of 55 481 individuals, we included those who provided data on abuse, self-harm and thoughts of suicide or self-harm on at least one occasion (*n* = 44 775). The sample was well-stratified across sociodemographic characteristics and all data were weighted to the proportions of gender, age, ethnicity, education and country of living obtained from the Office for National Statistics^11^ (see supplementary material available at https://doi.org/10.1192/bjp.2020.130 for further details on the sample and methods).

Thoughts of suicide or self-harm were assessed using the final item of the Patient Health Questionnaire (PHQ-9) (experiencing ‘thoughts that you would be better off dead or of hurting yourself in some way’). Self-harm was measured by asking participants if they had been ‘self-harming or deliberately hurting’. Physical abuse was measured by asking participants if they had been ‘physically harmed or hurt by someone else’. Psychological abuse was measured by asking if participants had been ‘bullied, controlled, intimidated, or psychologically hurt by someone else’. All responses were about the previous week and measured on a four-point scale from ‘not at all’ to ‘nearly every day’. For the analysis, we focused on any response that indicated any experience of self-harm or abuse on at least one occasion during the first month of lockdown.

Ethical approval for the COVID-19 Social Study was granted by the UCL Ethics Committee. All participants provided fully informed consent. The study is GDPR compliant.

## Results

Overall, 4121 participants (9%) surveyed reported experiencing psychological or physical abuse, 7984 participants (18%) reported experiencing thoughts of suicide or self-harm in the first month of lockdown and 2174 participants (5%) reported harming themselves at least once since the start of the UK's lockdown. For characteristics, see Table 1. Around 50% of participants experiencing psychological or physical abuse had experienced thoughts of suicide or self-harm, and 25% of them had engaged in self-harm behaviours during the previous week.

**Table 1:** Weighted sample characteristics (a) and mental health support strategies (b)^a^

	Total	Psychological abuse	Physical abuse	Self-harm/suicidal thoughts	Self-harm behaviours
*(a) Sample characteristics*	*N* = 44 775	*N* = 3736	*N* = 1317	*N* = 7984	*N* = 2174
Gender					
Female	22 846 (51.0%)	**2130 (9.3%)**	**530 (2.3%)**	4099 (17.9%)	1205 (5.3%)
Male	21 929 (49.0%)	**1605 (7.3%)**	**787 (3.6%)**	3885 (17.7%)	970 (4.4%)
Age, years					
18-29	7835 (17.5%)	**768 (9.8%)**	**304 (3.9%)**	**2270 (29.0%)**	**748 (9.6%)**
30-44	10 394 (23.2%)	**960 (9.2%)**	**325 (3.1%)**	**2102 (20.2%)**	**541 (5.2%)**
45-59	12 031 (26.9%)	**1106 (9.2%)**	**351 (2.9%)**	**2073 (17.2%)**	**549 (4.6%)**
60+	14 515 (32.4%)	**902 (6.2%)**	**337 (2.3%)**	**1540 (10.6%)**	**336 (2.3%)**
Ethnicity					
BAME groups	5259 (11.7%)	**656 (12.5%)**	**226 (4.3%)**	**1243 (23.6%)**	**401 (7.6%)**
White	39 516 (88.3%)	**3080 (7.8%)**	**1091 (2.8%)**	**6741 (17.1%)**	**1773 (4.5%)**
Marital status					
Living alone	9375 (20.9%)	**878 (9.4%)**	**344 (3.7%)**	**2208 (23.6%)**	**596 (6.4%)**
Not living with partner/spouse but living with another adult	9131 (20.4%)	**1072 (11.7%)**	**442 (4.8%)**	**2528 (27.7%)**	**823 (9.0%)**
Living with partner/spouse	26 269 (58.7%)	**1785 (6.8%)**	**531 (2.0%)**	**3248 (12.4%)**	**756 (2.9%)**
Children in the household					
No	33 197 (74.1%)	**2599 (7.8%)**	**932 (2.8%)**	**5987 (18.0%)**	**1606 (4.8%)**
Yes	11 578 (25.9%)	**1136 (9.8%)**	**385 (3.3%)**	**1997 (17.3%)**	**568 (4.9%)**
Employment status					
Employed	25 459 (56.9%)	**1985 (7.8%)**	**623 (2.4%)**	**3807 (15.0%)**	**889 (3.5%)**
Unemployed and seeking work	1504 (3.4%)	**221 (14.7%)**	**101 (6.7%)**	**564 (37.5%)**	**184 (12.2%)**
Not working (student/homemaker)	4625 (10.3%)	**488 (10.6%)**	**153 (3.3%)**	**1272 (27.5%)**	**392 (8.5%)**
Unable to work owing to disability	3124 (7.0%)	**574 (18.4%)**	**287 (9.2%)**	**1500 (48.0%)**	**556 (17.8%)**
Retired	10 063 (22.5%)	**468 (4.6%)**	**154 (1.5%)**	**841 (8.4%)**	**153 (1.5%)**
Educational attainment					
Degrees	15 167 (33.9%)	**1129 (7.4%)**	**227 (1.5%)**	**2156 (14.2%)**	**481 (3.2%)**
GCSE/A-levels	26 532 (59.3%)	**2379 (9.0%)**	**920 (3.5%)**	**5198 (19.6%)**	**1471 (5.5%)**
No qualifications	3076 (6.9%)	**228 (7.4%)**	**171 (5.6%)**	**630 (20.5%)**	**222 (7.2%)**
Income					
N-miss	4635	443	187	872	261
<£16 000	8324 (20.7%)	**1039 (12.5%)**	**482 (5.8%)**	**2345 (28.2%)**	**832 (10.0%)**
£16 000–29 999	11 165 (27.8%)	**884 (7.9%)**	**327 (2.9%)**	**1999 (17.9%)**	**483 (4.3%)**
£30 000–59 999	12 685 (31.6%)	**867 (6.8%)**	**217 (1.7%)**	**1828 (14.4%)**	**377 (3.0%)**
£60 000–89 999	4794 (11.9%)	**323 (6.7%)**	**65 (1.4%)**	**556 (11.6%)**	**123 (2.6%)**
>£90 000	3173 (7.9%)	**179 (5.6%)**	**39 (1.2%)**	**383 (12.1%)**	**97 (3.1%)**
Overcrowding					
No	42 853 (95.7%)	**3459 (8.1%)**	**1141 (2.7%)**	**7437 (17.4%)**	**1940 (4.5%)**
Yes	1922 (4.3%)	**276 (14.4%)**	**176 (9.1%)**	**546 (28.4%)**	**235 (12.2%)**
Mental health diagnosis					
No	36 018 (80.4%)	**2341 (6.5%)**	**821 (2.3%)**	**4171 (11.6%)**	**933 (2.6%)**
Yes	8757 (19.6%)	**1394 (15.9%)**	**496 (5.7%)**	**3813 (43.5%)**	**1241 (14.2%)**
Chronic physical illness					
No	27 588 (61.6%)	**2149 (7.8%)**	**724 (2.6%)**	**4679 (17.0%)**	**1261 (4.6%)**
Yes	17 187 (38.4%)	**1587 (9.2%)**	**593 (3.5%)**	**3305 (19.2%)**	**913 (5.3%)**
COVID-19 diagnosis					
No	**44 577 (99.6%)**	**3701 (8.3%)**	**1299 (2.9%)**	**7918 (17.8%)**	**2146 (4.8%)**
Yes	**198 (0.4%)**	**35 (17.6%)**	**18 (9.0%)**	**66 (33.4%)**	**28 (14.2%)**
Depressive symptoms (PHQ-9)					
Minimal/mild (0–9)	**31 585 (70.5%)**	**1444 (4.6%)**	**382 (1.2%)**	**1870 (5.9%)**	**340 (1.1%)**
Moderate (10–19)	**10 510 (23.5%)**	**1581 (15.0%)**	**613 (5.8%)**	**3908 (37.2%)**	**990 (9.4%)**
Severe (20+)	**2681 (6.0%)**	**710 (26.5%)**	**322 (12.0%)**	**2205 (82.3%)**	**845 (31.5%)**
Anxiety symptoms (GAD-7)					
Minimal/mild (0–10)	**35 440 (79.2%)**	**2035 (5.7%)**	**573 (1.6%)**	**3482 (9.8%)**	**752 (2.1%)**
Moderate (11–15)	**5297 (11.8%)**	**831 (15.7%)**	**378 (7.1%)**	**2080 (39.3%)**	**592 (11.2%)**
Severe (16+)	**4038 (9.0%)**	**870 (21.5%)**	**366 (9.1%)**	**2422 (60.0%)**	**830 (20.5%)**
Self-harm/suicidal thoughts					
Yes	7984 (17.8%)	**1932 (24.2%)**	**959 (12.0%)**	–	**1978 (24.8%)**
Self-harm behaviours					
Yes	2174 (4.9%)	**888 (40.8%)**	**718 (33.0%)**	**1978 (91.0%)**	–
Psychological/physical abuse					
Yes	4121 (9.2%)	–	–	**2154 (52.3%)**	**1011 (24.5%)**
Waves of data collection					
Mean (s.d.)	2.1 (1.1)	2.1 (1.1)	2.0 (1.1)	2.1 (1.1)	2.0 (1.1)
Range	1–5	1–5	1–5	1–5	1–5
*(b) Mental health support strategies*	*N* = 34 778)	*N* = 2873	*N* = 898)	*N* = 5881	*N* = 1508
Formal/structured support					
Taking medication (e.g. anti-depressants)	5534 (15.9%)	812 (28.3%)	309 (34.4%)	2157 (36.7%)	721 (47.8%)
Speaking with a mental health professional	908 (2.6%)	197 (6.8%)	94 (10.4%)	465 (7.9%)	219 (14.5%)
Speaking with a healthcare professional	495 (1.4%)	137 (4.8%)	72 (8.0%)	278 (4.7%)	115 (7.7%)
Speaking to somebody on a support helpline	189 (0.5%)	60 (2.1%)	20 (2.3%)	123 (2.1%)	69 (4.6%)
Online mental health programme (e.g. CBT)	321 (0.9%)	62 (2.2%)	24 (2.7%)	135 (2.3%)	60 (4.0%)
Any formal/structured support	6331 (18.2%)	990 (34.5%)	387 (43.1%)	2486 (42.3%)	858 (56.9%)
Informal support					
Online mental health forum	321 (0.9%)	83 (2.9%)	28 (3.1%)	182 (3.1%)	82 (5.4%)
Other mental health resources (e.g. self-help books, videos, or apps)	1489 (4.3%)	252 (8.8%)	70 (7.8%)	491 (8.3%)	160 (10.6%)
Mental health self-care (e.g. mindfulness or meditation)	7429 (21.4%)	882 (30.7%)	149 (16.6%)	1687 (28.7%)	392 (26.0%)
Speaking about mental health to a friend or family member	6644 (19.1%)	821 (28.6%)	230 (25.7%)	1915 (32.6%)	522 (34.6%)
Any informal support	10 951 (31.5%)	1323 (46.1%)	331 (36.9%)	2734 (46.5%)	723 (48.0%)

BAME, Black, Asian and minority ethnic; CBT, cognitive–behavioural therapy; N-miss, number of missing data.

a. Weighted sample characteristics: the values represent the percentage of participants who responded positively to each item. Values in bold in (a) represent statistically significant (*P* < 0.05) group comparisons (i.e. Psychological/physical abuse: No versus Yes; Suicidal/self-harm thoughts: No versus Yes; Self-harm behaviours: No versus Yes). *P*-values of group comparisons were calculated using chi-squared tests. Row percentages are showed in (a), whereas column percentages are shown in (b). The sample characteristics were calculated using data from the first wave of data collection for each participant. For abuse, self-harm and mental health support, we instead focused on any reporting during the first month of lockdown.

Data on mental health support were available for 34 778 individuals (Table 1). Around 60% of participants engaging in self-harm behaviours and 40% of participants with self-harm/suicidal thoughts or reporting abuse had accessed at least one type of formal or structured mental health support during the first month of lockdown (most commonly psychiatric medications). Almost 50% of participants in each group had used at least one type of informal mental health support such as talking with a friend or family member Table 1, supplementary Fig. 1).

## Discussion

Notably, the patterning of thoughts and experiences of self-harm and abuse reported during lockdown mirrored usual demographic characteristics, including being higher among younger adults, women, and people experiencing socioeconomic disadvantage, unemployment, disability, chronic physical illnesses and mental disorders.^5,7,9^ The elevated prevalence of abuse and self-harm thoughts/behaviours in people who had been diagnosed with COVID-19 could indicate a heightened psychological risk during infection, or increased risk of exposure due to either behavioural or occupational factors among individuals already self-harming. Comparisons with usual prevalence levels are challenging given that (i) our sample, though well-stratified and weighted to population proportions, was not random, (ii) underreporting of abuse remains likely, especially if people were living with their abuser during lockdown, (iii) our recruitment strategy involved partnership work with charities representing vulnerable people who may therefore have been more likely to report self-harm or abuse, and (iv) most prevalence data report on levels over a 12-month period or over a lifetime, whereas this study reported on a single month of lockdown. Therefore, the figures presented here are not intended to be accurate estimates of prevalence. Nevertheless, our data suggest that a substantial number of people were affected by these issues during lockdown.

Although 47.8% of people self-harming had been taking medication, only 14.5% had spoken with a mental health professional (lower than in previous prevalence studies).^3^ Similarly, only 7.9% of people experiencing thoughts of self-harm or suicide reported speaking with a mental health professional and 4.7% with another health professional (lower than the 25.5% reported in previous prevalence studies).^4^ Speaking with friends or family members was higher in our study (32.6%) than usually reported (21.7%).^4^ Levels of formal/structured help-seeking were lowest for people experiencing psychological abuse, and accessing informal support such as speaking with friends or family was lowest among people experiencing physical abuse.

Directly assessing prevalence of abuse, self-harm and thoughts of suicide or self-harm and how these compare with usual levels is challenging during the COVID-19 pandemic. In particular, the results presented in this study do not take into account the frequency or intensity of thoughts and experiences of self-harm and abuse. Further, our abuse measures did not ask about other types of abuse, such as sexual or financial, and numerous other risk factors were not considered. Nevertheless, the data presented here suggest that a substantial number of people experienced difficulties in the first month of lockdown, with fewer than half accessing either formal/structured or informal support. Hence, it is vital that new ways of identifying and evaluating individuals at risk of abuse and self-harm are swiftly rolled-out and additional support is made available to people at home.

## Data Availability

Data will be made publicly available following the end of the pandemic.

## Supplementary material

For supplementary material accompanying this paper visit http://dx.doi.org/10.1192/bjp.2020.130. click here to view supplementary material
