# Supplementary material for: Abuse, self-harm and suicidal ideation in the UK during the COVID-19 pandemic
Source: Br J Psychiatry. 2020 Jul 13:1–4. doi: 10.1192/bjp.2020.130 (PMC7360935; doi:10.1192/bjp.2020.130)
Supplement: Supplementary file 1 [file S0007125020001300sup.zip › S0007125020001300sup001.docx]

Supplementary Materials

Sample

The COVID-19 Social Study is a large panel study of the psychological and social experiences of over 75,000 adults (aged 18+) in the UK during the COVID-19 pandemic. The study commenced on 21^st^ March 2020 and involves online weekly data collection from participants for the duration of the COVID-19 pandemic in the UK. The study is not random and therefore is not representative of the UK population. However, it contains a well-stratified sample that was recruited using three primary approaches. First, snowballing was used, including promoting the study through existing networks and mailing lists (including large databases of adults who had previously consented to be involved in health research across the UK), print and digital media coverage, and social media. Second, more targeted recruitment was undertaken focusing on (i) individuals from a low-income background, (ii) individuals with no or few educational qualifications, and (iii) individuals who were unemployed. Third, the study was promoted via partnerships with third sector organisations to vulnerable groups, including adults with pre-existing mental health conditions, older adults, carers, and people experiencing domestic violence or abuse. The study was approved by the UCL Research Ethics Committee [12467/005] and all participants gave informed consent.

Measures

*Psychological abuse* was measured using a question asking whether the participant had experienced in the last week “being bullied, controlled, intimidated, or psychologically hurt by someone else”. *Physical abuse* was measured using a question asking whether the participant had experienced in the last week “being physically harmed or hurt by someone else”. For both questions, responses were rated on a 4-point scale ranging from “not at all” to “nearly every day”. We focused on any response that indicated any experience of psychological abuse on at least one occasion during the first month of lockdown (21^st^ March - 20^th^ April 2020).

*Thoughts of Death or Self-Harm* were measured using a specific item within the Patient Health Questionnaire (PHQ-9). This item asked whether the participant had experienced “thoughts that you would be better off dead or of hurting yourself in some way” in the last week. Responses were rated on a 4-point scale ranging from “not at all” to “nearly every day”. We focused on any response that indicated having such thoughts on at least one occasion during the first month of lockdown.

*Self-harm* was assessed using a question that asked whether the participant had been “self-harming or deliberately hurting yourself” in the past week. Responses were rated on a 4-point scale ranging from “not at all” to “nearly every day”. We focused on any response that indicated any self-harm behaviour on at least one occasion during the first month of lockdown.

*Mental Health Support* was measured through a question that asked whether the participant had done any of the following activities to support their mental health during the past week: 1) Taken medication (e.g. anti-depressants); 2) Spoken with a psychiatrist, psychologist or other mental health professional; 3) Spoken with a GP or other healthcare professional about their mental health; 4) Spoken to somebody on a support helpline (e.g. Samaritans or NHS Volunteers); 5) Accessed an online mental health programme (e.g. CBT); 6) Spoken with others on an online mental health forum; 7) Used other mental health resources (e.g. self-help books, videos, or apps); 8) Spent time on self-care specifically to help their mental health (e.g. mindfulness, meditation, or planning time for hobbies or relaxation); 9) Spoken about their mental health to a friend or family member. We focused on any response that indicated using any type of mental health support at least once during the first month of lockdown.

*Depressive symptoms* during the past week were measured using the Patient Health Questionnaire (PHQ-9), a standard instrument for diagnosing depression in primary care^1^. The questionnaire involves nine items, with responses ranging from “not at all” to “nearly every day”. Higher overall scores indicate more depressive symptoms, with scores of 0-4 suggesting minimal depression, 5-9 suggesting mild depression, 10-14 suggesting moderate depression, 15-19 suggesting moderately severe depression, and scores of 20-27 suggesting severe depression^2^

*Anxiety symptoms* during the past week were measured using the Generalised Anxiety Disorder assessment (GAD-7), a well-validated tool used to screen and diagnose generalised anxiety disorder in clinical practice and research^3^. There are 7 items with 4-point responses ranging from “not at all” to “nearly every day”, with higher overall scores indicating more symptoms of anxiety. Categorical scales suggest that scores higher than 5 are mild, higher than 10 are moderate, and higher than 15 are severe^1^.

A full study protocol can be seen at [www.COVIDSocialStudy.org](http://www.COVIDSocialStudy.org)

References

1. Löwe B, Kroenke K, Herzog W, Gräfe K. Measuring depression outcome with a brief self-report instrument: sensitivity to change of the Patient Health Questionnaire (PHQ-9). Journal of Affective Disorders 2004;81(1):61–6.

2. Kroenke K, Spitzer RL. The PHQ-9: A New Depression Diagnostic and Severity Measure. Psychiatr Ann 2002;32(9):509–15.

3. Spitzer RL, Kroenke K, Williams JB, Löwe B. A brief measure for assessing generalized anxiety disorder: the GAD-7. Archives of internal medicine 2006;166(10):1092–1097.

4. Overview of the UK population: November 2018. Office for National Statistics, 2018 (https://www.ons.gov.uk/releases/overviewoftheukpopulationnovember2018).


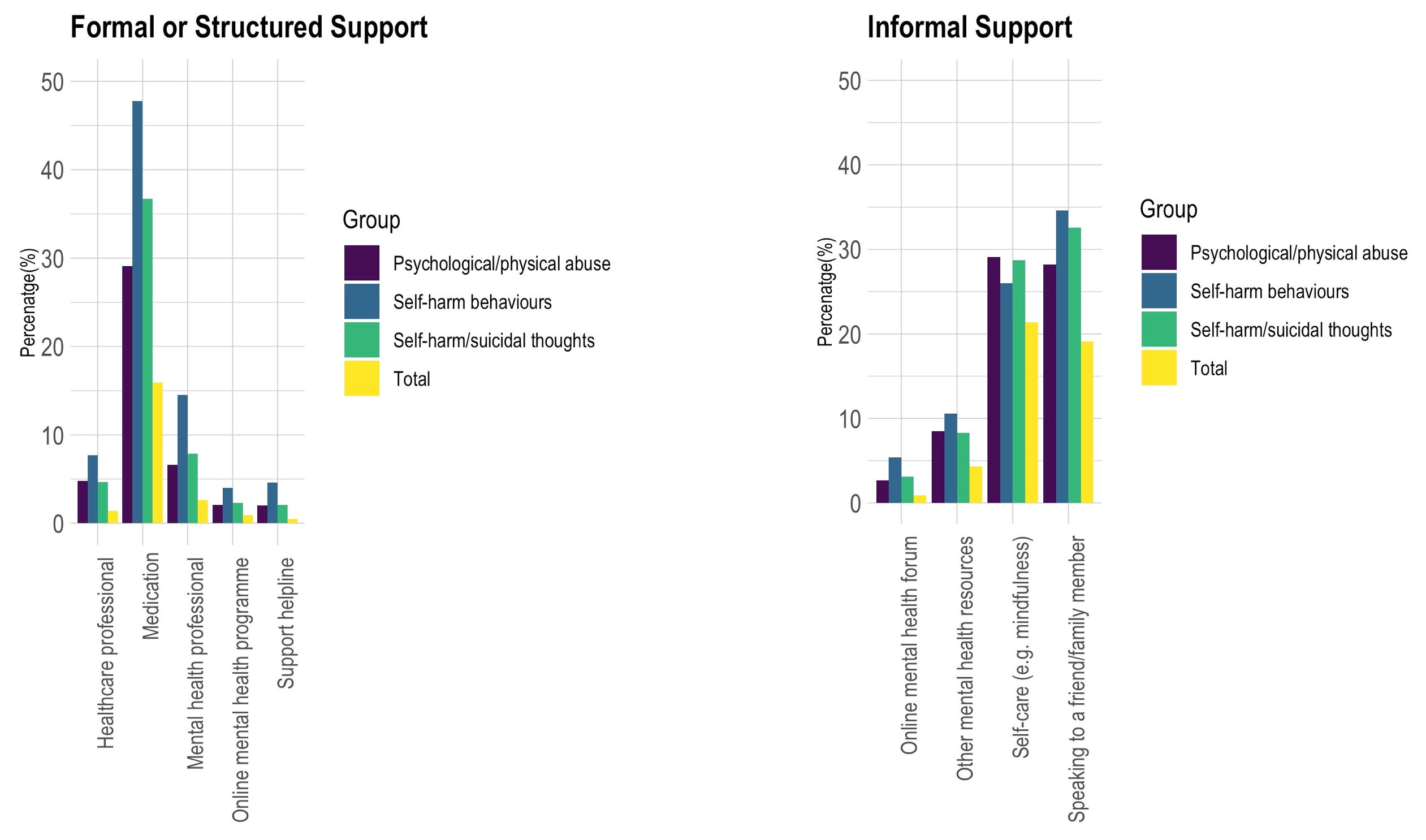


**sFigure1. Formal/Structured and Informal Mental Health Support by Abuse, Self-harm, and Thoughts of Death or Self-harm (weighted percentages).**
